# Psychosocial health in pregnancy and postpartum among women living with - and without HIV and non-pregnant women living with HIV living in Nordic countries – Results from a longitudinal survey study

**DOI:** 10.1186/s12884-021-04357-5

**Published:** 2022-01-07

**Authors:** Ellen Moseholm, Inka Aho, Åsa Mellgren, Gitte Pedersen, Terese L. Katzenstein, Isik S. Johansen, Diana Bach, Merete Storgaard, Nina Weis

**Affiliations:** 1 Department of Infectious Diseases, Copenhagen University Hospital, Kettegård Alle 30, 2650 Hvidovre, Hvidovre Denmark; 2 Department of Public Health, Faculty of Health and Medical Sciences, University of Copenhagen, Copenhagen, Denmark; 3 Department of Infectious Diseases, Helsinki University Hospital, Helsinki, Finland; 4 Department of Infectious Diseases, Region Vestra Gotland, Sahlgrenska University Hospital, Gothenburg, Sweden; 5 Department of Infectious Diseases, Institute of Biomedicine, Sahlgrenska Academy, University of Gothenburg, Gothenburg, Sweden; 6 Department of Infectious Diseases, Aalborg University Hospital, Aalborg, Denmark; 7 Department of Infectious Diseases, Copenhagen University Hospital, Rigshospitalet, Copenhagen, Denmark; 8 Department of Infectious Diseases, Odense University Hospital, Odense, Denmark; 9 Department of Gynecology and Obstetrics, Copenhagen University Hospital, Hvidovre, Hvidovre Denmark; 10 Department of Infectious Diseases, Aarhus University Hospital, Aarhus, Denmark; 11 Department of Clinical Medicine, Faculty of Health and Medical Sciences, University of Copenhagen, Copenhagen, Denmark

**Keywords:** Psychosocial health, depression, pregnancy, postpartum, women with HIV, 2BMOM

## Abstract

**Background:**

The success of antiretroviral therapy has normalized pregnancy among women living with HIV (WWH) with a very low risk of perinatal transmission of HIV. Despite these advances, WWH still face complex medical and psychosocial issues during pregnancy and postpartum. The aim of this study was to assess differences in psychosocial health outcomes between pregnant WWH, non-pregnant WWH, and pregnant women without HIV, and further identify factors associated with probable depression in the third trimester and postpartum.

**Methods:**

In a longitudinal survey study, participants were included from sites in Denmark, Finland, and Sweden during 2019–2020. Data was collected in the 3rd trimester, 3 and 6 months postpartum using standardized questionnaires assessing depression, perceived stress, loneliness, and social support. Mixed regression models were used to assess changes over time within and between groups. Logistic regression models were used to identify factors associated with depression in pregnancy and postpartum.

**Results:**

A total of 47 pregnant WWH, 75 non-pregnant WWH, and 147 pregnant women without HIV were included. The prevalence of depression was high among both pregnant and non-pregnant WWH. There was no significant difference between pregnant and non-pregnant WWH in depression scores, perceived stress scores, or social support scores at any time point. Compared to pregnant women without HIV, pregnant WWH reported worse outcomes on all psychosocial scales. Social support and loneliness were associated with an increased odds of depressive symptoms in the adjusted analysis.

**Conclusions:**

A high burden of adverse psychosocial outcomes was observed in both pregnant and non-pregnant women living with HIV compared to pregnant women without HIV. Loneliness and inadequate social support were associated with increased odds of depression in pregnancy and should be a focus in future support interventions.

**Supplementary Information:**

The online version contains supplementary material available at 10.1186/s12884-021-04357-5.

## Background

The success of combination antiretroviral therapy (cART) has resulted in a dramatic decrease in perinatal transmission of human immunodeficiency virus (HIV) to less than 1% in most Western countries, in addition to a normalization of pregnancy [1, 2]. This has resulted in an increase in the annual number of HIV pregnancies, both internationally and in Nordic countries [1, 3, 4]. However, despite these advancements, women living with HIV (WWH) still face complex psychosocial and medical issues during pregnancy, childbirth, and postpartum [5, 6].

Pregnancy and the postpartum period are times of significant biological, social, and psychological changes for a woman and HIV adds another layer of complexity [7–9]. For WWH, pregnancy may be a time of increased psychological vulnerability due to different contextual factors, disclosure issues, and HIV-related stigma [6, 9, 10]. Moreover, in addition to the usual stresses of new motherhood, WWH must also cope with stressors that include their own health, the unknown infectious states of their infants, and attending to their infants´ unique needs such as the administration of prophylactic antiretroviral medication [6, 11].

Psychosocial and emotional well-being during pregnancy is essential to develop the capacity to parent effectively and facilitate attachment to the new-born [12]. It is also well documented that maternal mental health during pregnancy and postpartum has a big impact child development and behaviour [13]. Research conducted in North America and in low- and middle-income settings [14] have reported a high prevalence of depression in pregnant and postpartum WWH [14–16]. A recent meta-analysis reported a significantly increased odds ratio of both antenatal and postnatal depressive symptoms in WWH compared to women without HIV (WWOH) [17]. Depression is one of the most common distress conditions in pregnancy and may be associated with the physical and emotional wellbeing of the mother, in addition to a range of adverse behavioural and emotional outcomes for the child [18]. For WWH adverse mental health outcomes may also affect HIV disease management during pregnancy or postpartum [14]. In a recent meta-analysis by Zhu et al. [17], optimal ART adherence was reported in 76% of pregnant and 53% of postpartum WWH worldwide, and depression and emotional stress has been  identified as barriers to adherence [19].

The broader social and environmental context may be important determinants for mental health problems during pregnancy and postpartum. Previous research among WWOH have reported a significant association between poor maternal social support and depressive symptoms in pregnancy [20, 21] and the postpartum period [22]. WWH have been found to have lower levels of social support when compared to WWOH [23], and research among pregnant WWH have shown that perceived stress and social isolation may be important factors associated with higher levels of depression [24–26].

There is a lack of research exploring WWH’s varied experiences of pregnancy and motherhood in a Western context, where medical systems allow for appropriate medical treatment to all WWH.

Using quantitative data from the 2BMOM Study, a multicentre longitudinal mixed methods study among pregnant WWH, non-pregnant WWH, and pregnant WWOH in the Nordic countries Denmark, Finland, and Sweden [27], we aim to explore psychosocial health outcomes of WWH across the pregnancy–postpartum trajectory, assess differences in psychosocial health outcomes between pregnant WWH, non-pregnant WWH and pregnant WWOH, and identify factors associated with depression in the third trimester and postpartum for WWH and WWOH.

## Methods

### Setting

There are approximately 1600, 1000, and 2800 WWH in Denmark, Finland, and Sweden, respectively [4, 28, 29]. The majority of WWH in Scandinavia are immigrants, mainly from sub-Saharan Africa, and mainly infected with HIV by sexual contact [4, 30]. The healthcare system in the Nordic countries is tax-based and ensures universal access to medical health care, including antenatal, perinatal, and postpartum care, and many social support services [31]. cART is provided free of charge and people with HIV in Nordic countries are generally well treated with life expectancies approaching those of the general population [4, 29, 32].

### The 2BMOM study

The 2BMOM study is a multicenter longitudinal mixed-methods study investigating psychosocial outcomes and experiences of WWH in Nordic countries during pregnancy and early motherhood. The study recruited pregnant WWH, non-pregnant WWH, and pregnant WWOH from seven sites in Denmark, Finland, and Sweden between January 2019 and December 2020. Quantitative data was collected by self-administered electronic questionnaires, with a sub-sample of pregnant WWH taking part in semi-structured qualitative interviews (*n* = 31). Methods are described in detail elsewhere [27]. All women gave informed consent to participate and the study was approved by the Danish Data Protection Agency (VD-2018-253), and the Finnish and Swedish Ethics Committees (HUS/1330/2019 and Dnr: 2019–04451, respectively).

### Study population

Pregnant and non-pregnant WWH were consecutively recruited from the participating sites (Departments of Infectious Diseases at Copenhagen University Hospitals, Hvidovre and Rigshospitalet; Odense -,Aalborg – and Aarhus University Hospitals in Denmark, Department of Infectious Diseases, Helsinki University Hospital, Finland, and Department of Infectious Diseases, Sahlgrenska University Hospital, Sweden) by the medical staff during routine clinical appointments. Pregnant WWH were asked to participate if they were 18 years of age or older, pregnant, anticipated birth of a viable infant without life-threatening conditions or congenital anomalies, and could speak and read Danish, Finnish, Swedish or English. Non-pregnant WWH were asked to participate if they were between 18 and 45 years of age, not pregnant or planning to become pregnant, and could speak and read Danish, Finnish, Swedish, or English. Pregnant WWOH were consecutively recruited from the Department of Obstetrics at Copenhagen University Hospital, Hvidovre, Denmark, which has the largest maternity ward in Denmark with approximately 7000 deliveries per year, and the Department of Obstetrics at Helsinki University Hospital, Finland. Women were asked to participate if they were 18 years of age or older, pregnant, not living with HIV, anticipated birth of a viable infant without life-threatening conditions or congenital anomalies, had no known chronic or psychiatric illness associated with increased surveillance or adverse pregnancy and birth outcome, and could speak and read Danish, Finnish or English.

### Data collection

Participants answered electronic standardized questionnaires via REDCap© at three time points; in the 3rd trimester, 3 and 6 months postpartum (T1, T2, and T3, respectively). Non-pregnant WWH followed the same timeframe, i.e. baseline following enrolment, 3–4 months, and 6–7 months. At each timepoint, a survey link was sent to the participants, who then completed the survey at home.

### Demographics and medical information

The demographic variables were collected at baseline (T1) and included self-reported marital status, education, and employment status. Information on clinical variables (maternal country of birth, maternal age at delivery, parity, CD4 cell count, HIV RNA viral load, co-morbidity, cART, non-HIV medication, smoking, alcohol, and drug use) was obtained from the patients’ medical records.

### Survey instrument

Information on different psychosocial health outcomes were collected using the following validated scales:

#### Depression

The Edinburgh Postnatal Depression Scale (EPDS) was used to assess symptoms of perinatal depression [33]. The scale has 10-items with responses on a 4-point Likert scale ranging from 0 (absence of depressive moods) to 3 (worst mood). A total score ranging from 0 to 30 is calculated, and a cut-off point of ≥12 indicates an increased likelihood of clinical depression [33]. The EPDS has been translated into Danish, Finnish, and Swedish, has a well-documented validity and reliability, and has been shown to be sensitive to changes over time [33, 34]. The scale does not mention the words pregnancy, child, birth or infant, and has also been validated in a non-pregnant population [35, 36].

#### Perceived stress

Perceived stress was measured using the Perceived Stress Scale – 10 item (PSS-10), a self-administered scale that was developed to measure “the degree to which situations in one’s life are appraised as stressful” during the past month [37]. Responses are on a Likert-type five-point format that ranges from 0 (never) to 4 (very often). The range for scores is 0 to 40 with higher scores reflecting greater perceptions of stress. The PSS-10 has been translated and validated in Danish, Finnish, and Swedish [38–40].

#### Social isolation

Perceived social isolation was assessed using the short version of the 3rd version of the University of California, Los Angeles (UCLA) loneliness scale [41, 42]. The scale consists of three items with Likert scale responses ranging from 1 (hardly ever) to 3 (often). Responses are summed into a total score (ranging from 3 to 9), where higher scores indicate a greater degree of loneliness [41, 42]. A score > 7 has been suggested as a cut-off for severe loneliness [43]. The UCLA loneliness scale is a widely used self-report scale measuring loneliness, with acceptable validity and reliability [41, 44]. The scale has been translated and validated in a Danish and Finnish context, respectively [45, 46]. The scale was translated into Swedish using a translation–back-translation procedure before data collection [47].

#### Social support

The Multidimensional Scale of Perceived Social Support (MSPSS), a 12–item scale, was used to assess social support [48, 49]. The scale consists of three subscales; Family, Friends, and Significant others. Each item is answered from 1 (strongly disagree) to 5 (strongly agree). A total score ranging from 1 to 7 is calculated, with higher scores suggesting a greater level of perceived social support. The MSPSS is a frequently used measure of social support in somatic illness, and good reliability and validity have been reported, also in a Scandinavian context [50–53].

### Statistical analysis

Differences in baseline demographic characteristics were summarized and compared between pregnant WWH, and non-pregnant WWH and pregnant WWOH, respectively, using the Pearson’s χ2-test or Student’s unpaired T-test, as appropriate. Categorical variables were described as counts (%), and continuous variables were described as means (95% confidence intervals (CI)). Linear mixed effects models for repeated measures were used to assess changes in mean scores of the different psychosocial health outcomes (depression, stress, loneliness, and social support) over time and between groups. These models account for correlations between repeated measurements of the same women over time and also include all available data, i.e. if a woman had missing data at one time point they were not deleted from the analysis, and the available data from previous time points was included resulting in more precise estimates. Within-subject residuals were modeled with an unstructured variance-covariance structure. All mixed models were bootstrapped with 2000 repetitions to account for the nonparametric distribution of data. Univariate and multivariate logistic regression models were completed to identify factors associated with probable depression in pregnancy and postpartum, respectively. This analysis was restricted to pregnant WWH and pregnant WWOH with a pregnancy depression score (*n* = 45 and *n* = 146) and a postpartum depression score (*n* = 36 and *n* = 122). Probable depression was defined using the validated cut-off score on the EPDS scale (score ≥ 12). The variables included in the multivariate model were chosen based on the results from the univariate analysis using a stepwise selection to choose the most parsimonious model. The final model selection was based on Akaike’s Information Criterion. All models included living with HIV, perceived stress, loneliness, and social support in pregnancy, relationship status, country of birth, and education. Unknown/other/missing categories were included in the analysis. The model with probable depression postpartum as the dependent variable also included depression score in pregnancy. Analyses were performed using STATA 17 software and all reported *p*-values are two-sided using a significance level of 0.05.

## Results

In total, 71 pregnant WWH fulfilled the inclusion criteria during the study period; 57 agreed to participate. However, six women did not complete the baseline survey before delivery, two had a preterm delivery, and two women withdrew their consent. Thus, a total of 47 pregnant WWH were included in the study giving a response rate of 66%. The main reasons for non-participation were language barriers and major psychiatric or social complications. The number of pregnant WWH who also completed the follow-up surveys T2 and T3 were 38 (80%) and 37 (79%), respectively. A total number of 112 non-pregnant WWH agreed to participate, of which 75 (67%) completed T1 and were thus included, of these 50 (68%) women completed T2 and 49 (65%) completed T3. A total number of 168 pregnant WWOH agreed to participate, of which 147 (88%) completed T1, 132 (89%) completed T2, and 125 (85%) completed T3. Enrollment and data collection were for some women conducted during the COVID-19 pandemic (see Supplementary 1).

### Characteristics

The demographics of the study population are presented in Table 1. Compared to non-pregnant WWH, pregnant WWH were significantly younger, were more likely to be married or living with a partner, less likely to be employed part or full time, had fewer comorbidities, and higher viral loads at inclusion. None of the participants used illicit drugs, and none of the pregnant populations had any alcohol use. Two non-pregnant WWH had an increased use of alcohol. All pregnant WWH were virally suppressed at the time of delivery. Compared to pregnant WWOH, pregnant WWH were significantly more likely to be of non-Nordic origin,  less likely to be married or living with a partner, to have a high level of education, to be employed part or full time, to be nulliparous, and more likely to deliver by a planned cesarean section. A larger proportion of WWH also had a preterm delivery.

**Table 1:** Baseline characteristics

	WWH	Non-pregnant WWH	Pregnant WWOH	***p***-value
	(*n* = 47)	(*n* = 75)	(*n* = 147)	
**Age, mean (95% CI)**	33.65 (32.14: 35.15)	36.01 (34.45: 37.57)	32.25 (31.57: 33.93)	0.04*
**Relationship status, n (%)**				< 0.01***
Married/living with a partner	36 (76)	39 (52)	142 (97)	
Have a partner, but not living together	5 (11)	11 (15)	0	
Do not have a current partner	4 (9)	23 (31)	5 (3)	
Unknown/prefer not to say	<3 (4)	<3 (2)	0	
**Country of birth, n (%)**				< 0.001**
Nordic country (Denmark; Finland or Sweden)	12 (26)	31 (41)	135 (91)	
Asia	0	4 (5)	0	
Africa	25 (53)	27 (36)	< 3 (1)	
Eastern Europe/Russia	6 (13)	8 (11)	0	
Other	4 (8)	5 (7)	11 (7)	
**Education, n (%)**				< 0.001**
Primary school	3 (6)	6 (8)	3 (2)	
Secondary school	13 (28)	18 (24)	14 (10)	
Higher education (college/university)	27 (57)	50 (67)	129 (87)	
Unknown/Missing	4 (9)	< 3 (1)	< 3 (1)	
**Employment, n (%)**				< 0.01***
Yes, part or full time	29 (62)	61 (81)	126 (86)	
**Smoking, n (%)**				0.09
During pregnancy	< 3 (2)	7 (9)	0	
Missing	0	< 3 (3)	8 (5)	
**Comorbidities, n (%)**	6 (13)	24 (32)	21 (14)	0.02*
**Number of children, n (%)**				< 0.001**
0	18 (38)	26 (35)	85 (58)	
1	18 (38)	21 (28)	57 (39)	
≥ 2	11 (24)	28 (37)	5 (3)	
**Years since HIV diagnosis, mean (95% CI)**	9.45 (7.28: 11.61)	11.58 (10.04: 13.13)		0.10
**HIV diagnosis in pregnancy, n (%)**				
Yes	3 (6)			
**Mode of HIV transmission, n (%)**				0.71
Sexual	42 (89)	65 (87)		
Perinatal transmission	5 (11)	6 (8)		
Unknown/other	0	4 (5)		
**ART treatment**^**+**^**, n (%)**				0.36
NRTIs + NNRTI	11 (24)	10 (13)		
NRTIs + PI	16 (34)	22 (29)		
NRTIs + InSTI	18 (38)	39 (52)		
Other	<3 (4)	4 (5)		
**CD4 cell count**^**+**^**, n (%)**				0.12
> 500 cells/mL	36 (76)	66 (88)		
200–500 cells/mL	6 (13)	7 (9)		
< 200 cells/mL	5 (11)	<3 (3)		
**HIV viral load**^**+**^**, n (%)**				0.02*
< 50 copies/mL	39 (83)	73 (97)		
> =50 copies/mL	8 (17)	< 3 (3)		
**Mode of delivery, n (%)**				< 0.01**
Vaginal delivery	32 (68)		104 (71)	
Elective caesarean delivery	9 (19)		7 (5)	
Acute caesarean delivery	4 (9)		19 (13)	
Unknown/missing	<3 (4)		17 (11)	
**Complications during delivery, n (%)**	12 (26)		36 (24)	0.58
Unknown/missing	7 (15)		21 (14)	
**Gestational age < 37 weeks, n (%)**	4 (9)		< 3 (1)	0.03**

*P* values are based on the Pearson’s χ2-test, Fischers exact or unpaired T-test, as appropriate

*Significant difference between pregnant WWH and non-pregnant WWH

**Significant difference between pregnant WWH and pregnant WWOH

***Significant difference between pregnant WWH and non-pregnant WWH, and significant difference between pregnant WWH and pregnant WWOH

^+^At time of inclusion

*WWH* Women living with HIV, *WWOH*: Women without HIV

### Difference in psychosocial outcomes over time and between groups

**Fig. 1:**
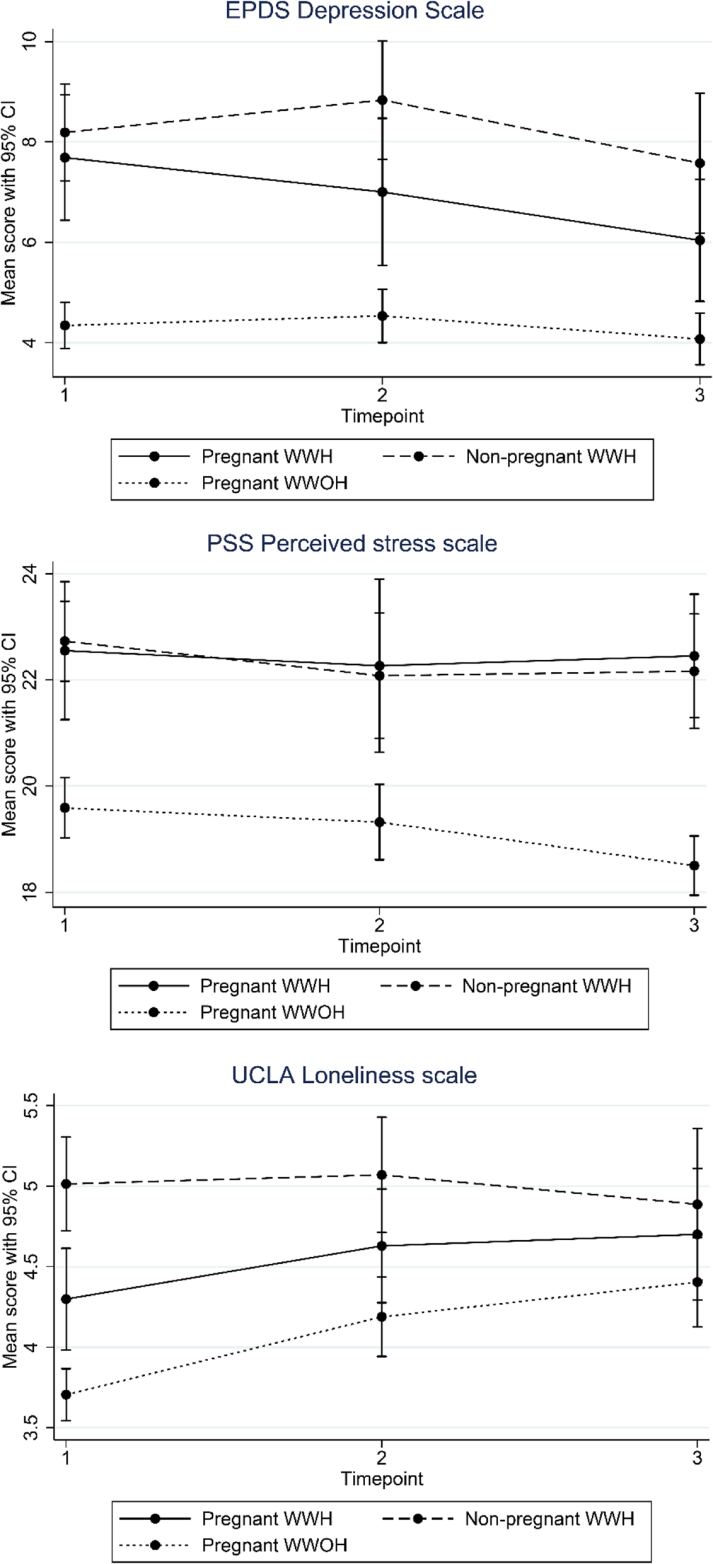
Depression, Perceived stress and Loneliness among pregnant women living with HIV (WWH), non-pregnant WWH, and pregnant women without HIV (WWOH) over time. Figure legend: EPDS Depression scale: A higher score = more depressive symptoms. PSS Perceived stress scale: A higher score = more perceived stress. UCLA Loneliness scale: A higher score = more loneliness

**Fig. 2:**
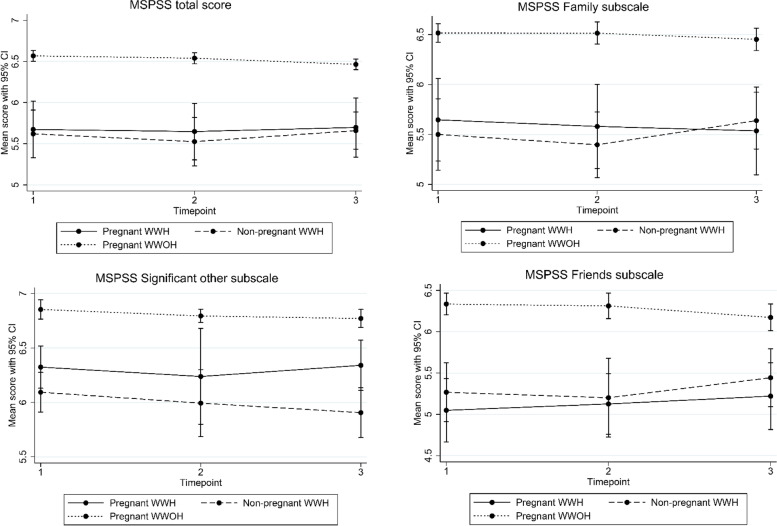
Social support measured by the Multidimensional Scale of Perceived Social Support (MSPSS) scale among pregnant women living with HIV (WWH), non-pregnant WWH, and pregnant women without HIV (WWOH) over time. Figure Legend: MSPSS: A higher score = more social support

The mean scores of the psychosocial health outcomes for each group and at each time point are presented in Table 2, Figs. 1 and 2.

**Table 2:** Psychosomatic health outcome at T1-T3 between pregnant women living with HIV (WWH), non-pregnant women living with HIV (WWH) and pregnant women without HIV (WWOH)

	Range	Pregnant women with HIV			Non-pregnant women with HIV			Pregnant women without HIV			Difference between pregnant and non-pregnant WWH			Difference between pregnant WWH and pregnat WWOH		
		T1	T2	T3	T1	T2	T3	T1	T2	T3	T1	T2	T3	T1	T2	T3
**Edinburg depression scale (EPDS)**		*n* = 45	*n* = 36	*n* = 36	*n* = 74	*n* = 49	*n* = 46	*n* = 146	*n* = 130	*n* = 122	*p-value*	*p-value*	*p-value*	*p-value*	*p-value*	*p-value*
Total score, mean (95% CI)	0–30	7.68 (6.49: 8.93)	7.00 (5.53: 8.47)	6.04 (4.82: 7.25)	8.19 (7.22: 9.15)	8.83 (7.65: 10.01)	7.58 (6.18: 8.97)	4.34 (3.89: 4.79)	4.53 (4.12: 4.94)	4.07 (3.56: 4.57)	0.53	0.06	0.10	**< 0.01**	**< 0.01**	**< 0.01**
Likely clinical depression (score ≥ 12), n (%)		11 (24)	13 (36)	8 (22)	14 (27)	20 (41)	17 (39)	13 (9)	11 (8)	10 (8)						
**Perceived Stress (PSS)**		*n* = 44	*n* = 36	*n* = 36	*n* = 73	*n* = 49	*n* = 46	*n* = 146	*n* = 131	*n* = 124						
Total score, mean (95% CI)	0–40	22.55 (21.24: 23.45)	22.26 (20.63: 23.89)	22.45 (21.28: 23.61)	22.73 (21.97: 23.50)	22.08 (20.90: 23.26)	22.16 (21.08: 23.24)	19.59 (19.04: 20.15)	19.32 (18.61: 20.03)	18.50 (17.94:19.02)	0.80	0.86	0.74	**< 0.001**	**< 0.01**	**< 0.001**
Low stress (score 0–13), n (%)		2 (5)	1 (3)	1 (3)	2 (3)	1 (2)	1 (2)	7 (5)	3 (2)	9 (7)						
Moderate stress (score 14–26), n (%)		33 (75)	29 (81)	29 (81)	52 (71)	39 (80)	38 (83)	128 (88)	125 (95)	112 (90)						
High stress (score 27–40), n (%)		9 (20)	6 (17)	6 (17)	19 (26)	9 (18)	7 (15)	11 (8)	3 (2)	3 (2)						
**Loneliness (UCLA)**		*n* = 44	*n* = 36	*n* = 36	*n* = 72	*n* = 47	*n* = 46	*n* = 146	*n* = 130	*n* = 123						
Total score, mean (95% CI)	3–9	4.30 (3.98: 4.61)	4.63 (4.28: 4.98)	4.70 (4.29: 5.11)	5.01 (4.72: 5.31)	5.07 (4.71: 5.43)	4.89 (4.42: 5.36)	3.71 (3.54:3.87)	4.19 (3.94: 4.44)	4.40 (4.13: 4.68)	**< 0.01**	0.11	0.59	**< 0.01**	0.05	0.19
Lonely (score > 7), n (%)		0	2 (6)	3 (8)	8 (11)	3 (6)	6 (13)	1 (1)	6 (5)	8 (6)						
**Social support (MSPSS)**		*n* = 44	*n* = 36	*n* = 36	*n* = 72	*n* = 47	*n* = 46	*n* = 146	*n* = 130	*n* = 123						
Total score, mean (SD)	1–7	5.67 (5.33: 6.02)	5.65 (5.30: 5.99)	5.70 (5.43: 5.98)	5.62 (5.33: 5.91)	5.53 (5.23: 5.82)	5.67 (5.43: 5.89)	6.57 (6.37: 6.63)	6.54 (6.47: 6.61)	6.46 (6.40: 6.53)	0.83	0.61	0.87	**< 0.001**	**< 0.001**	**< 0.001**
Significant other, mean (SD)	1–7	6.32 (6.13: 6.52)	6.24 (5.80: 6.68)	6.34 (6.11: 6.57)	6.09 (5.91: 6.28)	5.99 (5.69: 6.30)	5.91 (5.68: 6.14)	6.85 (6.77: 6.94)	6.79 (6.73: 6.85)	6.77 (6.69: 6.85)	0.10	0.31	0.06	**< 0.01**	**0.02**	**< 0.001**
Family, mean (SD)	1–7	5.65 (5.23: 6.06)	5.58 (5.16: 6.00)	5.54 (5.10: 5.97)	5.50 (5.14: 5.86)	5.40 (5.07: 5.73)	5.64 (5.35: 5.92)	6.52 (6.42: 6.61)	6.51 (6.40: 6.62)	6.45 (6.33: 6.56)	0.65	0.53	0.73	**< 0.001**	**0.001**	**< 0.01**
Friends, mean (SD)	1–7	5.05 (4.68: 5.43)	5.13 (4.76: 5.49)	5.22 (4.82: 5.62)	5.27 (4.91: 5.62)	5.20 (4.73: 5.68)	5.44 (5.09: 5.79)	6.33 (6.20: 6.46)	6.31 (6.16: 6.47)	6.17 (6.01: 6.33)	0.47	0.77	0.50	**< 0.001**	**< 0.001**	**< 0.001**

The mean EPDS score among pregnant WWH was in the third trimester 7.68 (95% CI: 6.49: 8.93). There was a significant decrease in depression scores over time among pregnant WWH (mean difference between T1 and T3 EPDS score: -1.65 (95% CI: − 2.99: − 0.30), *p*-value = 0.02). Using the recommended cutoff, the prevalence of probable depression among pregnant WWH was 24% in the third trimester, 36% at 3 months postpartum, and 22% at 6 months postpartum. There was no difference in depression scores or perceived stress scores between pregnant and non-pregnant WWH at any time point. Pregnant WWH reported significantly higher depression scores and more perceived stress at all time points compared to pregnant WWOH. Pregnant WWOH reported significantly less stress over time (mean difference between T1 and T3 PSS score: -1.09 (95% CI: − 1.73: − 0.44), *p*-value = < 0.01) while there was no change in perceived stress in any of the WWH groups.

Pregnant WWH reported significantly lower scores on the UCLA loneliness scale at T1 compared to non-pregnant WWH and significantly higher loneliness scores at T1 compared to pregnant WWOH. However, over time there was a significant increase in loneliness scores among pregnant WWH (mean difference between T1 and T3 UCLA score: 0.46 (95% CI: 0.06: 0.85), *p*-value = 0.03) and among pregnant WWOH over time (mean difference between T1 and T3 UCLA score: 0.70 (95% CI: 0.41: 0.98), *p*-value = < 0.001). Thus, the proportion of women with a loneliness score above the cut-off increased over time, although the absolute numbers are low. There was no significant difference in loneliness scores between pregnant and non-pregnant WWH, and between pregnant WWH and pregnant WWOH at T2 and T3 (Fig. 1).

Pregnant and non-pregnant WWH reported similar MSPSS social support scores over time and there was no significant difference within or between groups (Fig. 2).

Compared to the pregnant WWOH, pregnant WWH at all times and in all sub-scales reported significantly less social support (Fig. 2). Pregnant WWOH reported significantly lower support from friends over time (mean difference between T1 and T3 MSPSS friends sub-scale: -0.16 (95% CI: − 0.28: − 0.05), *p*-value = 0.03).

### Factors associated with probable depression

The results of the univariate and multivariate analysis are presented in Table 3. In the univariate analysis, living with HIV, perceived stress, loneliness, social support, being born in a non-Nordic country, having a primary/secondary education and not being employed were associated with increased odds of probable depression in pregnancy. When adjusting for HIV status, different maternal characteristics, and pregnancy psychosocial outcomes, only loneliness score in pregnancy remained significantly associated with increased odds of probable depression in pregnancy (OR 1.56 (95% CI: 1.01: 2.41), *p*-value 0.04) while social support in pregnancy was associated with a decreased odds of probable depression in pregnancy (OR 0.50 (95% CI: 0.27: 0.95), *p*-value 0.03).

**Table 3:** Logistic regression analysis of factors associated with probable depression in pregnancy and depression postpartum among pregnant women living with HIV and pregnant women without HIV

	Depression in pregnancy				Depression postpartum			
	***Univariate***		***Adjusted***^**a**^		***Univariate***		***Adjusted***^**a**^	
	OR (95% CI)	*p-value*	OR (95% CI)	*p-value*	OR (95% CI)	*p-value*	OR (95% CI)	*p-value*
**Living with HIV**	2.83 (1.19: 6.71)	**0.02**	0.75 (0.14; 4.17)	0.75	3.2 (1.16: 8.85)	**0.03**	2.39 (0.34: 16.76)	0.38
**Pregnancy depression score (EPDS score)**					4.55 (1.47: 14.07)	**< 0.01**	1.99 (0.46: 8.73)	0.92
**Stress (PSS score)**	1.15 (1.05: 1.27)	**< 0.01**	1.23 (1.00: 1.27)	0.05	1.20 (1.06: 1.35)	**< 0.01**	1.14 (0.96: 1.34)	0.14
**Loneliness (UCLA score)**	2.37 (1.67: 3.35)	**< 0.001**	1.56 (1.01: 2.41)	**0.04**	1.62 (1.23: 2.15)	**< 0.01**	1.58 (1.08: 2.31)	**0.02**
**Social support (MSPSS score)**	0.34 (0.22: 0.54)	**< 0.001**	0.50 (0.27: 0.95)	**0.03**	0.40 (0.24: 0.67)	**< 0.001**	0.58 (0.28: 1.19)	0.14
**Age**	1.03 (0.22: 0.54)	0.57			0.93 (0.82: 1.05)	0.22		
**Relationship status**								
Married/living with a partner	ref		ref		ref		ref	
Have a partner, but not living together	2.33 (0.23: 23.43)	0.47	0.57 (0.04: 7.92)	0.68	4.13 (0.35: 48.08)	0.26	3.20 (0.02: 449.36)	0.65
Do not have a current partner/prefer not to say	2.63 (0.65: 10.65)	0.18	0.65 (0.06: 6.74)	0.72	1.38 (0.16: 12.16)	0.78	1.00 (0.09: 11.33)	0.99
**Country of birth**								
Nordic country (Denmark; Finland or Sweden)	ref		ref		ref		ref	
Not Nordic country	3.19 (1.31: 7.79)	**0.01**	1.16 (0.20: 0.86)	0.87	2.54 (0.87: 7.46)	0.09	0.28 (0.03: 2.56)	0.26
**Education**								
Higher education (college/university)	ref		ref		ref			
Primary/secondary school	3.5 (1.43: 8.59)	**< 0.01**	1.74 (0.53: 5.63)	0.36	2.06 (0.67: 6.34)	0.21	0.31 (0.05: 1.74)	0.18
**Employment**								
Yes, part or full time	0.30 (0.12: 0.73)	**< 0.01**			0.81 (0.25: 2.68)	0.73		
**Number of children**								
0	ref				ref			
≥ 1	1.97 (0.84: 4.59)	0.12			0.95 (0.35: 2.55)	0.92		
**Mode of delivery**								
Vaginal delivery	ref				ref			
Caesarean delivery	1.02 (0.35: 2.99)	0.97			0.87 (0.23: 3.27)	0.83		
**Preterm (gestational age < 37 weeks)**	3.35 (0.58: 19.32)	0.18			–			

^a^Adjusted for living with HIV, perceived stress, loneliness, and social support in pregnancy, relationship status, country of birth, and education. The postpartum depression model also included depression score in pregnancy

Factors associated with increased odds of probable postpartum depression were in the univariate analysis living with HIV, depression score in pregnancy, postpartum perceived stress, loneliness, and social support. Only postpartum loneliness remained significantly associated with probable postpartum depression in the adjusted analysis (OR 1.58 (95% CI 1.08: 2.31), *p*-value 0.02).

## Discussion

Using quantitative data from the 2BMOM study [27] this study found a high prevalence of probable depression and other psychosocial challenges among WWH compared to WWOH throughout the pregnancy – postpartum trajectory. There were no significant differences in depression scores, perceived stress scores, or social support scores between pregnant WWH and non-pregnant WWH at any time point. Loneliness was significantly associated with increased odds of probable depression in pregnancy and postpartum, while social support was associated with a decreased odds of probable depression in pregnancy.

Both pregnant and non-pregnant WWH had a high prevalence of probable depression (24% and 27% at T1, respectively) compared to pregnant WWOH (9% at T1). A high prevalence of depression among both pregnant and non-pregnant WWH is well described in the literature [15, 17, 23, 54, 55]. Pregnant WWH reported significantly higher depression scores at all time points compared to pregnant WWOH. This is contrary to findings from several US-based studies reporting no difference in depression scores between pregnant WWH and WWOH [15, 23, 56]. However, in these studies depressive symptoms were overall high, making it difficult to detect significant differences between groups.

Pregnant WWH had significantly lower depression scores over time. Pregnancy and becoming a mother may have a beneficial effect on women’s mental health and may act as a buffer to some of the negative effects of living with HIV [6, 57, 58]. A small study among WWH in Spain found that although pregnancy was associated with more negative emotions such as anxiety, fear, guilt, and sadness compared to WWOH, pregnancy was also associated with positive emotions, such as happiness [57]. This is supported by other studies, where motherhood is found to be one factor that lightens the burden of living with HIV [6, 58, 59]. We also found that pregnant WWH reported significantly lower loneliness scores in pregnancy compared to non-pregnant WWH.

Loneliness and perceived social support have been described as major risk factors for depression in pregnancy and postpartum in both WWH and WWOH [25, 60]. In our analysis, loneliness and low social support were significantly associated with increased odds of probable depression in pregnancy, while postpartum loneliness was significantly associated with increased odds of probable postpartum depression. Living with HIV was not significantly associated with depression in the multivariate analysis, suggesting that social and/or psychological factors and not HIV per se may be the main contributor to depression. Although not significant in the multivariate analysis, stress was associated with increased odds of probable depression in pregnancy. Living with HIV can contribute to women’s experience of shame, exclusion, and rejection from family, partners, and even healthcare providers [6, 58, 61]. This can lead to an increased feeling of isolation if support is limited, in addition to stress related to lack of control or limited feeling of resilience [25].

Both WWH and pregnant WWOH reported an increase in loneliness scores postpartum. This is in line with other studies [25, 60], suggesting that the postpartum period may be a period of increased parenting responsibility and limited social activities which may increase the feeling of loneliness. Moreover, WWH experienced lower social support on all domains compared to pregnant WWOH. Studies among pregnant and postpartum WWH have reported an increased risk of depression with poorer social support [23, 54]. Pregnant WWH were more likely to not be married or living with a partner than pregnant WWOH. Research among WWOH suggests that increases in both partner and family support may be powerful protective factors for decreasing mental health difficulties in pregnancy and postpartum, highlighting the importance of targeting and increasing this type of support from pregnancy to the postpartum period [62]. Other supportive conditions for perinatal wellbeing include having access to high-quality information on HIV and being ensured the same rights as other women [63].

To our knowledge, this is the first study examining psychosocial health across the pregnancy – postpartum trajectory among pregnant WWH compared to pregnant WWOH and non-pregnant WWH. Hence, the results of this study provide a comprehensive knowledge of the psychosocial experiences and emotional care needs of WWH in a Western setting. Another strength is the use of validated scales to assess the different psychosocial outcomes. Although we included a nationwide sample of pregnant WWH in Denmark, in addition to a sample of WWH in both Finland and Sweden, the small number of pregnant WWH and the fact that they all live in Nordic countries may limit the generalizability of the results. The main reason for non-participation were language barriers and major psychiatric or social complications. Hence, a vulnerable group of women were not included, and the results may therefore reflect women with more resilience. The participants were followed longitudinally allowing us to capture changes in experiences over time. However, there was a gradual loss to follow-up over time, which further limited our sample. Enrollment and data collection was for some women conducted during the COVID-19 pandemic, which could potentially have influenced our findings in a more adverse direction [64]. However, the proportion of women who completed the survey during the COVID-19 pandemic were similar across groups, and the within-group change over time was limited and consistent with the literature.

## Conclusion

Understanding and responding to the experiences of pregnant and postpartum women living with HIV is important so that goal-oriented interventions supporting these women can be developed. Women living with HIV reported more depressive symptoms, perceived stress, and loneliness, and less social support across the pregnancy – postpartum trajectory compared to pregnant women without HIV. Compared to non-pregnant women living with HIV, pregnant women living with HIV were less lonely while pregnant. The prevalence of probable depression was high among both pregnant and non-pregnant women living with HIV. Loneliness and inadequate social support are important factors associated with an increased odds of depressive symptoms during pregnancy and should be a focus in future support interventions.

## Supplementary Information

**Additional file 1:** **Supplementary 1**. Number of women who completed the survey during the COVID-19 pandemic (after 1 March 2020) by group and timepoint

## Data Availability

The data that support the findings of this study are available upon approval from the relevant Regulatory agencies (the Danish Data Protection Agency and the Finnish and Swedish Ethics Committees), but restrictions apply to the availability of these data, which were used under license for the current study, and so are not publicly available. Data are however available from the authors upon reasonable request and with permission of the Danish Data Protection Agency, and the Finnish and Swedish Ethics Committees.

## Abbreviations

cART: Combination Antiretroviral Therapy
EPDS: The Edinburgh Postnatal Depression Scale
HIV: Human Immunodeficiency Virus
MSPSS: The Multidimensional Scale of Perceived Social Support
PSS-10: Perceived Stress Scale – 10 item
UCLA Loneliness Scale: University of California, Los Angeles (UCLA) loneliness scale
WWH: Women living with HIV
WWOH: Women without HIV
