# Supplementary material for: Psychosocial health in pregnancy and postpartum among women living with - and without HIV and non-pregnant women living with HIV living in the Nordic countries – Results from a longitudinal survey study
Source: BMC Pregnancy Childbirth. 2022 Jan 7;22:20. doi: 10.1186/s12884-021-04357-5 (PMC8740861; doi:10.1186/s12884-021-04357-5)
Supplement: Supplementary file 1 — Additional file 1: Supplementary 1. Number of women who completed the survey during the COVID-19 pandemic (after 1 March 2020) by group and timepoint [file 12884_2021_4357_MOESM1_ESM.docx]

**Supplementary 1**

Number of women who completed the survey during the COVID-19 pandemic (after 1 March 2020) by group and timepoint.

|  | **Pregnant WWH** | **Non-pregnant WWH** | **Pregnant WWOH** |
| --- | --- | --- | --- |
|  | n=47 | n=75 | n=168 |
|  |  |  |  |
| Timepoint | n (%) | n (%) | n (%) |
| T1 | 13 (28) | 25 (33) | 0 |
| T2 | 22 (47) | 25 (33) | 18 (11) |
| T3 | 29 (62) | 34 (45) | 72 (43) |

WWH: women living with HIV

WWOH: women without HIV
